# Prognostic and predictive impact of NOTCH1 mutations in patients with chronic lymphocytic leukemia: a tertiary single-center experience

**DOI:** 10.3389/fonc.2025.1726439

**Published:** 2026-01-13

**Authors:** Mattia D’Antiga, Andrea Serafin, Francesco Angotzi, Alessandro Cellini, Arianna Bevilacqua, Giovanni Leone, Nicolò Danesin, Chiara Adele Cavarretta, Francesco Piazza, Erich Piovan, Laura Bonaldi, Livio Trentin, Andrea Visentin

**Affiliations:** 1 Hematology Unit, Department of Medicine, University of Padua, Padua, Italy; 2 Immunology and Molecular Oncology Unit, Veneto Institute of Oncology IOV-IRCCS, Padua, Italy; 3 Department of Surgery, Oncology and Gastroenterology, University of Padua, Padua, Italy

**Keywords:** CLL, NOTCH1, overall survival, prognostic factors, target therapy

## Abstract

NOTCH1 mutations (NOTCH1^m^) occur in 6%–12% of newly diagnosed chronic lymphocytic leukemia (CLL) patients, increasing to 15%–20% in relapsed cases. Despite their clinical relevance, the independent prognostic impact of NOTCH1^m^ remains controversial, particularly in the era of targeted therapies, and routine testing has not been universally adopted. A retrospective, real-world study of 271 consecutive CLL patients treated at our institution was conducted between 1999 and 2023. The association of NOTCH1^m^ with clinical outcomes and response to different treatment modalities, including chemoimmunotherapy (CIT), Bruton’s tyrosine kinase inhibitors (BTKi), and venetoclax-based regimens, was evaluated. Primary endpoints included time to first treatment (TTFT), time to second treatment (TT2T), time to next treatment (TTNT), and overall survival (OS). NOTCH1^m^ were detected in 38/271 (14%) patients, predominantly the c.7541_7542delCT deletion (84%). After a median follow-up of 118 months, NOTCH1^m^ patients demonstrated significantly shorter OS compared to *NOTCH1* wild-type (NOTCH1^wt^) patients (244 vs. 293 months, HR=1.92, p=0.032), but this was not confirmed in a Cox multivariate analysis, where immunoglobulin heavy-chain variable region (IGHV) resulted as the independent prognostic variable. Importantly, 44% of Richter transformation cases harbored NOTCH1^m^. Among NOTCH1^m^ patients, targeted therapies showed superior TT2T compared to CIT (NR vs. 48 months, p=0.024). No significant difference was observed in TTFT or TTNT between NOTCH1^m^ and wild-type patients. In conclusion, NOTCH1^m^ are associated with adverse prognosis in CLL, primarily due to increased risk of Richter transformation. Our findings support incorporating *NOTCH1* mutational analysis into routine clinical practice for improved risk stratification and treatment selection.

## Introduction

Chronic lymphocytic leukemia (CLL) is the most common adult leukemia in Western countries, characterized by the clonal proliferation and accumulation of mature B lymphocytes in peripheral blood, bone marrow, and lymphoid tissues. The clinical course of CLL is remarkably heterogeneous and shaped by chromosomal aberrations and recurrent gene mutations such as *TP53*.

The *NOTCH1* gene encodes a class I transmembrane protein functioning as a ligand-activated transcription factor. The mature NOTCH1 receptor is a heterodimer whose intracellular domain (NOTCH1-ICD) contains a PEST domain that regulates stability and proteasomal degradation of the active protein. *NOTCH1* signaling starts with the release of NOTCH1-ICD by the sequential activity of *ADAM* and gamma-secretase at the cell membrane, leading to the downstream activation of target genes, including *c-MYC*^2^. In CLL, NOTCH1^m^ occur in approximately 6%–12% of newly diagnosed cases (1), increasing to 15%–20% in progressive or relapsed patients and up to 30% in Richter transformation (RT) (2). The most common mutation is a 2-base-pair frameshift deletion (c.7541_7542delCT) in exon 34, accounting for approximately 80% of NOTCH1^m^ in CLL (3). This generates a premature stop codon, causing the truncation of the C-terminal PEST domain (P2514fs*4), resulting in a failed proteasomal degradation and leading to aberrantly prolonged signaling.

Data concerning the independent prognostic impact of the NOTCH1^m^ in CLL are conflicting, with some retrospective and prospective studies reporting a shorter overall survival (OS) (4, 5) while in others, they were not found to have an impact on either time to first treatment (TTFT) (6) or progression-free survival (PFS) (5, 7, 8).

## Methods

In this study, we aimed to comprehensively evaluate the prognostic and predictive impact of NOTCH1^m^ in a retrospective, real-world cohort of 271 consecutive CLL patients treated at our institution between 1999 and 2023 in whom biological samples for *NOTCH1* mutational analysis were available. Patients with no NOTCH1^m^ detected were referred to as *NOTCH1* wild-type (NOTCH1^wt^) in the context of this paper. We assessed the association of NOTCH1^m^ with clinical outcomes and response to different treatment modalities, chemoimmunotherapy (CIT), continuous therapy with Bruton’s tyrosine kinase inhibitors (BTKi), and time-limited venetoclax (VEN)-based regimens.

Baseline characteristics and laboratory findings were collected from medical records. NOTCH1^m^ analysis in the C-terminal PEST domain was performed through the combination of Sanger sequencing and ARMS PCR for the *NOTCH1* c.7541_7542delCT mutation (estimated limit of detection 1%–5%; representative results available in **Supplementary Figure 2**) on peripheral blood or bone marrow samples collected at diagnosis, treatment initiation, and disease progression. Treatment decisions were made according to the international workshop on CLL (iwCLL) guidelines and institutional protocols based on the standard of care at the time of treatment initiation. Patients were categorized into three main treatment groups: CIT, BTKi, and VEN-based. Response assessment was performed according to the 2018 iwCLL criteria. The primary endpoints were TTFT, time to second treatment (TT2T), time to next treatment (TTNT), and OS. OS was estimated using the Kaplan–Meier method and compared across groups using the log-rank test. Univariate and multivariate Cox proportional hazards regression models were used to assess the prognostic impact of NOTCH1^m^, immunoglobulin heavy-chain variable region (IGHV) mutational status, treatment era, *TP53* mutations, age at diagnosis, and complex karyotype on OS. Missing data were handled using multiple imputation by chained equations (MICE) with 10 imputations. Results from complete case analysis and MICE were compared to assess the robustness of findings, with complete case analysis presented as the main result. The proportional hazards assumption was assessed using Schoenfeld residuals. All statistical tests were two-sided, with p < 0.05 considered statistically significant. Analyses were performed using R version 4.5.2 with the *{survival}*, *{survminer}*, and *{mice}* packages. Potential collinearity between covariates was assessed using Spearman’s rank correlation coefficients. Categorical variables were compared using chi-square or Fisher’s exact test as appropriate. Continuous variables were compared using the Mann–Whitney U test for non-normally distributed data. Survival curves were estimated using the Kaplan–Meier method and compared using the log-rank test. The association between NOTCH1^m^ and treatment response was evaluated separately for each treatment category and within the context of the therapy era, defined as treatment/diagnosis before and after 2015, to capture the differences between treatment with CIT and targeted therapy.

All patients provided written informed consent for participation in the study (4430/AO/18), which was approved by the local ethics board on 27 June 2019 and was conducted according to the principles of the Declaration of Helsinki and the Good Clinical Practice Guidelines.

## Results

Baseline characteristics of the population are detailed in **Table 1**. Overall, NOTCH1^m^ were detected in 38/271 (14%) patients (c.7541_7542delCT in 84% of cases; full list in **Supplementary Table 1**), NOTCH1^m^ were detected in 27 (9.9%) at diagnosis, and 11 (4.1%) acquired NOTCH1^m^ at relapse. Two NOTCH1^m^ patients at diagnosis resulted in wild type at relapse. Five patients carried both *NOTCH1* and *TP53* mutations at diagnosis, while one patient acquired both mutations at relapse. NOTCH1^m^ patients displayed significantly more splenomegaly (52.8% vs. 29.5%; p=0.010, **Table 1**) and bulky lymphadenopathy (22.2% vs. 9.1%; p=0.038); conversely, NOTCH1^wt^ harbored more mutated IGHV (23.7% vs. 54.0%, p=0.001).

**Table 1:** Baseline characteristics according to NOTCH1 mutational status.

Characteristic	NOTCH1 wild-type (n=233)	NOTCH1 mutated (n=38)	P-value
Demographics			
Male sex, n (%)	132/232 (56.9)	20/38 (52.6)	0.753
Age at diagnosis, years	61.9 ± 11.0	63.8 ± 11.5	0.330^*^
Age at treatment initiation, years	66.2 ± 10.4	66.0 ± 10.7	0.950^*^
Clinical features			
B symptoms, n (%)	10/232 (4.3)	1/38 (2.6)	1.000^†^
Palpable splenomegaly, n (%)	65/220 (29.5)	19/36 (52.8)	**0.01**
Bulky lymphadenopathy >5 cm, n (%)	20/220 (9.1)	8/36 (22.2)	**0.038^†^**
Laboratory parameters			
Hemoglobin, g/dL	13.6 [12.7–14.6]	14.0 [13.2–14.6]	0.441^‡^
White blood cell count, ×10^9^/L	14.9 [10.9–24.9]	19.0 [13.5–27.1]	0.152^‡^
Platelet count, ×10^9^/L	191 [157–232]	215 [154–254]	0.273^‡^
Molecular characteristics			
Unmutated IGHV, n (%)	86/187 (46.0)	29/38 (76.3)	**0.001**
Cytogenetic abnormalities			
TP53 mutations, n (%)	30/228 (13.2)	6/35 (17.1)	0.596^†^
del(17p), n (%)	24/230 (10.4)	6/38 (15.8)	0.401^†^
del(11q), n (%)	33/229 (14.4)	4/38 (10.5)	0.698
del(13q14.3), n (%)	136/230 (59.1)	19/38 (50.0)	0.38
Trisomy 12, n (%)	45/230 (19.6)	9/38 (23.7)	0.713
Complex karyotype (>5 abnormalities), n (%)	31/190 (16.3)	3/30 (10.0)	0.495^†^
Treatment characteristics			
Untreated patients, n (%)	80/233 (34.3)	11/38 (28.9)	0.641
Relapsed patients, n (%)	79/233 (33.9)	16/38 (42.1)	0.424
Number of treatment lines	1.0 [0.0–2.0]	1.0 [0.0–2.0]	0.370^‡^
Diagnosis/treatment before 2015, n (%)	133/233 (57.1)	21/38 (55.3)	0.973
First-line treatment			
Chemoimmunotherapy, n (%)	113/154 (73.4)	16/27 (59.3)	0.206
BTK inhibitors, n (%)	28/154 (18.2)	8/27 (29.6)	0.266
Ibrutinib, n (%)	22/28 (78.6)	7/8 (87.5)	1.000^†^
Acalabrutinib, n (%)	6/28 (21.4)	1/8 (12.5)	1.000^†^
Venetoclax regimens, n (%)	13/154 (8.4)	3/27 (11.1)	0.712^†^
Second-line treatment			
Chemoimmunotherapy, n (%)	53/78 (67.9)	10/16 (62.5)	0.896
BTK inhibitors, n (%)	17/78 (21.8)	3/16 (18.8)	1.000^†^
Venetoclax regimens, n (%)	5/78 (6.4)	2/16 (12.5)	0.330^†^
PI3Kδ inhibitors, n (%)	3/78 (3.8)	1/16 (6.2)	0.532^†^
Disease transformation			
Richter syndrome, n (%)	10/233 (4.3)	7/38 (18.4)	**0.004^†^**

Data are presented as mean ± SD, median [IQR], or n (%).

BTK, Bruton tyrosine kinase; IQR, interquartile range; IGHV, immunoglobulin heavy-chain variable region.

^†^Fisher’s exact test.

^*^Student’s t-test.

^‡^Wilcoxon–Mann–Whitney test.

bold values indicate statistically significant p values.

No difference in demographics, laboratory parameters, cytogenetic abnormalities, and treatment characteristics was observed. The possible confounding role of treatment era was studied through the distribution of first-line treatment types stratified by treatment era and NOTCH1 mutational status (n=178), with no significant difference in CIT and targeted treatments between NOTCH1^m^ and NOTCH1^wt^ (Pearson’s chi-squared test with Yates’ continuity correction: p=0.175), while understandably, they differed significantly between eras (Fisher’s exact test: p < 0.001) (**Supplementary Table 4**).

**Figure 1:**
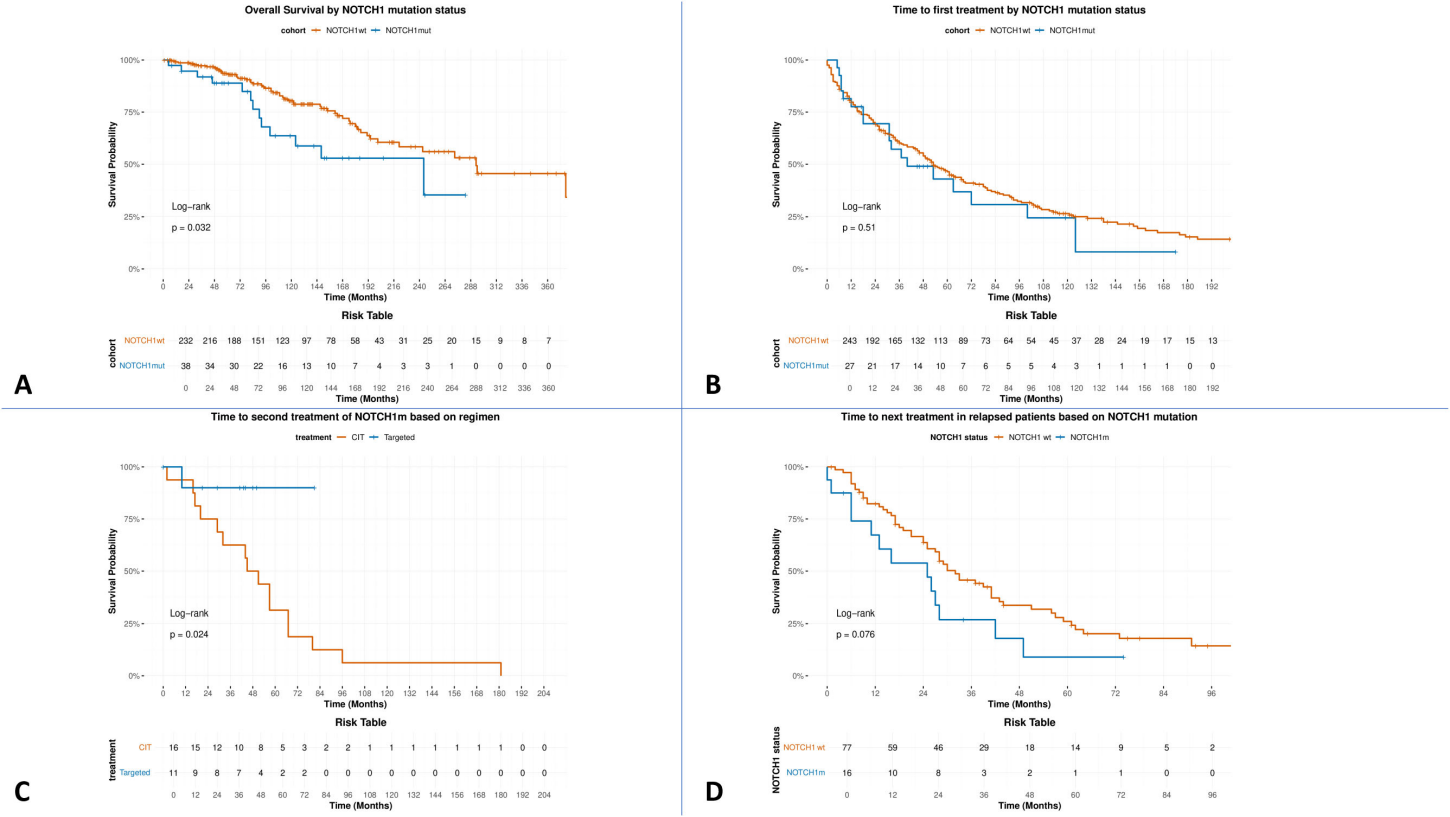
Impact of NOTCH1 mutation status on key clinical outcomes (A) Kaplan-Meier curves showing overall survival (OS) stratified by NOTCH1 mutation status (B) Time to first treatment (TTFT) by NOTCH1 mutation status (C) Time to second treatment in NOTCH1-mutated patients stratified by treatment regimen (chemoimmunotherapy [CIT] vs targeted therapy). (D) Time to next treatment in relapsed/refractory patients stratified by NOTCH1 mutation status. NOTCH1mut: NOTCH1 mutations; NOTCH1wt: NOTCH1 wild-type.

After a median follow-up of 118 months, NOTCH1^m^ patients had significantly shorter OS (244 vs. 293 months, HR=1.92, 95% CI: 1.04–3.54, p=0.032) compared to NOTCH1^wt^ patients (**Figure 1A**). The overall survival at 120 months was 80.5% vs. 63.3%. A Cox univariate analysis was performed to detect significant variables on OS, and then a multivariate analysis was performed to detect the impact of significant parameters (**Supplementary Table 2**). In univariate analysis, unmutated IGHV (HR=2.94, 95% CI: 1.61–5.36, p < 0.001) and older age at diagnosis (HR=1.11 per year, 95% CI: 1.08–1.14, p < 0.001) were significantly associated with inferior OS, together with NOTCH1^m^ (HR=1.93, 95% CI: 1.05–3.55, p=0.035). In multivariate analysis, only unmutated IGHV (HR=2.48, 95% CI: 1.22–5.03, p=0.012) and age (HR=1.09 per year, 95% CI: 1.05–1.13, p < 0.001) remained independently prognostic in complete case analysis. NOTCH1^m^ did not show independent prognostic significance (HR=1.53, 95% CI: 0.69–3.38, p=0.29). Results were consistent between complete case analysis (n=180) and MICE analysis (n=270), with excellent concordance for IGHV status (2.7% difference in HR) and age (1.1% difference). The model demonstrated good discriminative ability (C-index=0.728), and overall model fit was significant (likelihood ratio test p < 0.001). However, the proportional hazards assumption was violated for treatment era (Schoenfeld test p=0.024), indicating that the effect of treatment era on survival changes over time. Consequently, treatment era was not independently prognostic in the multivariate model (HR=1.16, 95% CI: 0.59–2.26, p=0.67).

Potential interactions were tested between NOTCH1^m^ and IGHV status and between NOTCH1^m^ and treatment era. Neither interaction was statistically significant in the complete case analysis (NOTCH1 × IGHV: LRT χ^2^ = 0.002, p=0.97; NOTCH1 × treatment era: LRT χ^2^ = 0.001, p=0.98). To further explore potential subgroup-specific effects, stratified analyses examining NOTCH1^m^ were performed separately in IGHV-unmutated and IGHV-mutated patients. Among patients with complete *NOTCH1* and IGHV data (n=225), NOTCH1^m^ showed a trend toward worse survival in IGHV-mutated patients (HR=3.26, 95% CI: 0.89–11.94, p=0.075) but not in IGHV-unmutated patients (HR=1.29, 95% CI: 0.63–2.65, p=0.49). Analysis of four combined groups (**Supplementary Table 3**) demonstrated that compared to the reference group (NOTCH1^wt^/IGHV-mutated, median OS 377 months), both the NOTCH1^wt^/IGHV-unmutated (HR=3.25, p=0.0007) and NOTCH1^m^/IGHV-unmutated (HR=4.27, p=0.0008) groups showed significantly worse survival. The NOTCH1^m^/IGHV-mutated group showed a trend toward worse survival (HR=3.41, p=0.06), although this did not reach statistical significance, likely due to the small sample size (n=9). Global comparison across all four groups was significant (log-rank p=0.0009). Assessment of potential collinearity revealed minimal correlation between NOTCH1^m^ and other prognostic factors (Spearman’s ρ: NOTCH1^m^ vs. IGHV=0.15, NOTCH1^m^ vs. TP53 = 0.08, NOTCH1^m^ vs. age=−0.05).

RT was associated with NOTCH1^m^ (18.4% vs. 3.9%; p=0.003), with NOTCH1^m^ detected in 44% (7/16) patients who developed RT, but there was no difference in time to RT development based on NOTCH1^m^ (80 vs. 76 months, HR=0.94, 95% CI: 0.31–2.81, p=0.92). Competing risk analysis accounting for death as a competing event showed no significant difference in cumulative incidence of RT between groups (p=0.93). The cumulative incidence of RT at 100 months was 62.5% (95% CI: 18.4–87.8%) for NOTCH1^wt^ and 85.7% (95% CI: 12.9–98.8%) for NOTCH1^m^ (**Supplementary Figure 3**).

Patients harboring NOTCH1^m^ displayed similar TTFT compared with NOTCH1^wt^. The median TTFT was 40 vs. 53 months (HR=1.17, 95% CI: 0.72–1.88, p=0.51; **Figure 1B**). The 120-month TTFT was 26.5% vs. 24.5%.

No association in TT2T was observed between NOTCH1^m^ and NOTCH1^wt^ in different frontline treatments (**Supplementary Figure 1A**). With CIT, the median TT2T was 48 and 45 months for NOTCH1^m^ and NOTCH1^wt^, respectively (HR=1.16, 95% CI: 0.67–2.00, p=0.58). Instead, the median TT2T was not reached with BTKi and VEN. The 36-month TT2T with BTKi and VEN-based treatment was 87% vs. 77% (HR=0.47, 95% CI: 0.05–3.93, p=0.48) and 100% vs. 100% (HR NA, 95% CI: NA–NA, p=1) for NOTCH1^m^ and NOTCH1^wt^, respectively.

Among NOTCH1^m^ cases, we observed a significant benefit in TT2T for patients treated with a targeted therapy compared to CIT, NR vs. 48 months (HR=0.13, 95% CI: 0.02–1.04, p=0.024) (**Figure 1C**).

At first relapse (n=95), 63 patients were treated with CIT, 20 with BTKi, four with PI3Kδ inhibitors, and seven with VEN (**Table 1**). Among relapsed patients, NOTCH1^m^ determined a trend toward worse TTNT outcomes, 25 vs. 32 months (HR=1.72, 95% CI: 0.94–3.18, p=0.076) compared to unmutated patients (**Figure 1D**). Among NOTCH1^m^ relapsed patients (n=16), no difference in TTNT was detected between CIT and targeted regimens, 16 vs. 27 months, respectively (HR=0.98, 95% CI: 0.31–3.15, p=0.98).

Concerning the difference between CIT and targeted therapies, there was a trend toward an OS benefit for targeted agents as first line in NOTCH1^wt^ (NR vs. 243 months, HR=0.29, 95% CI: 0.07–1.21, p=0.07), which was not present in the NOTCH1^m^ cohort (NR vs. 148 months, HR=0.44, 95% CI: 0.05–3.66, p=0.44) (**Supplementary Figure 1B**).

## Discussion

In our cohort, the frequency of NOTCH1^m^ at diagnosis (9.9%) aligns with the 5%–10% typically reported in newly diagnosed patients with CLL (1). The predominance of the c.7541_7542delCT deletion confirms this as the main mutation hotspot, consistent with previous reports (3). The increasing frequency of NOTCH1^m^ with treatment exposure and disease progression has been previously reported (2) and suggests clonal selection under therapeutic pressure, consistent with *NOTCH1* signaling promoting cell survival and drug resistance. Our observation that 44% of cases with RT harbored NOTCH1^m^ represents one of the highest reported frequencies (9) and confirms the strong association between *NOTCH1* activation and histologic transformation, even though in our study it was not associated with a faster development of RT nor a difference in CI (10). This finding has important clinical implications for risk stratification and surveillance strategies, as these relapse/refractory patients may benefit from closed monitoring and/or specific surveillance.

Of interest are the cases of two patients who were NOTCH1^m^ at diagnosis but wild type at relapse, which may be due to clonal evolution with possible dominance of a different clone at relapse, with the original clone no longer detectable through Sanger sequencing.

In our cohort of treated CLL patients, NOTCH1^m^ were not independently prognostic for OS after adjusting for IGHV status and age. This contrasts with some prior reports suggesting NOTCH1^m^ as an adverse prognostic factor, but is consistent with more recent studies in the era of novel targeted agents.

The lack of independent prognostic value of NOTCH1^m^ may may be due to confounding by IGHV status, so we tested this hypothesis through additional testing: we found no evidence of a significant interaction between NOTCH1^m^ and IGHV status (NOTCH1 × IGHV: LRT χ^2^ = 0.002, p=0.97), suggesting that their prognostic effects are independent and additive rather than synergistic. Although stratified analysis showed a numerical trend toward a stronger NOTCH1^m^ effect in IGHV-mutated patients (HR=3.26 vs. 1.29), the wide confidence intervals and lack of statistical significance preclude definitive conclusions, especially given the low prevalence of NOTCH1^m^ in this subgroup (n=9 with 3 events). Ultimately, analysis of four combined groups based on NOTCH1 and IGHV status (**Supplementary Table 3**) demonstrated that unmutated IGHV was the dominant adverse prognostic factor independent of NOTCH1^m^. Larger cohorts are needed to definitively assess potential IGHV subgroup-specific effects of NOTCH1^m^.

Similarly, we found no evidence that treatment era modifies the prognostic impact of NOTCH1^m^ (interaction p=0.98), suggesting that in our real-life cohort, novel targeted agents do not differentially benefit NOTCH1^m^ compared to NOTCH1^wt^ cases. However, the proportional hazards assumption was violated for treatment era (p=0.024), indicating that the relative benefit of novel agents changes over time. This time-dependent effect warrants further investigation in larger cohorts with extended follow-up.

In our population, NOTCH1^m^ were not a determining factor in TTFT (6), and the worst outcomes for OS align with previous older studies (4, 5), but an independent prognostic role was not demonstrated in our cohort of patients exposed also to targeted agents. The total absence of even a trend toward improved OS in NOTCH1^m^ treated with targeted agents in first line warrants further investigation. Interestingly, *TP53* and high-count Complex Karyotype (CK) were also not associated with worse OS outcomes in our real-world population (11, 12). Our results in time to second treatment align with a recent meta-analysis that demonstrated a shorter TTNT (8) in patients with NOTCH1^m^, even though secondary analysis of a pivotal clinical trial with BTKi and VEN-based therapy did not confirm this association (13–16). However, some studies have demonstrated delayed lymph node shrinkage and higher residual disease rates in patients with NOTCH1^m^ after BTKi (17) and VEN-based therapy (15), respectively.

Despite the prognostic significance of NOTCH1^m^ and its potential role in treatment selection, routine testing for these mutations has not been universally adopted in clinical practice (18). This may be due to several factors, including the lack of standardized testing protocols, uncertainty about the optimal timing of testing, and limited data on how NOTCH1^m^ status should influence treatment decisions in the era of targeted therapies.

Our study has several strengths, including the long follow-up period, comprehensive molecular characterization, and inclusion of patients treated with modern targeted therapies. The real-world nature of our cohort enhances the applicability of our findings to routine clinical practice. However, several limitations should be acknowledged: the retrospective design introduces potential selection bias, and treatment allocation was not randomized. The relatively low prevalence of NOTCH1^m^ in the treated population (15%, 27/179) limited statistical power to detect moderate effects, particularly in treatment subgroup analyses. The violation of the proportional hazards assumption for treatment era suggests that time-dependent models may be more appropriate for assessing temporal changes in treatment efficacy.

In conclusion, NOTCH1^m^ were associated with a negative prognostic impact, likely due to the development of RT rather than response to treatments. Our results suggest that analysis of NOTCH1^m^ should be incorporated in clinical practice.

## Data Availability

The raw data supporting the conclusions of this article will be made available by the authors, without undue reservation.

## Supplementary material

Supplementary Figure 1 **(A)** time to second treatment based on NOTCH1 status and first line regimen **(B)** Overall survival based on NOTCH1 mutation status and regimen.
