# Supplementary figures and images for: Prognostic and predictive impact of NOTCH1 mutations in patients with chronic lymphocytic leukemia: a tertiary single-center experience
Source: Front Oncol. 2026 Jan 13;15:1726439. doi: 10.3389/fonc.2025.1726439 (PMC12834786; doi:10.3389/fonc.2025.1726439)

A

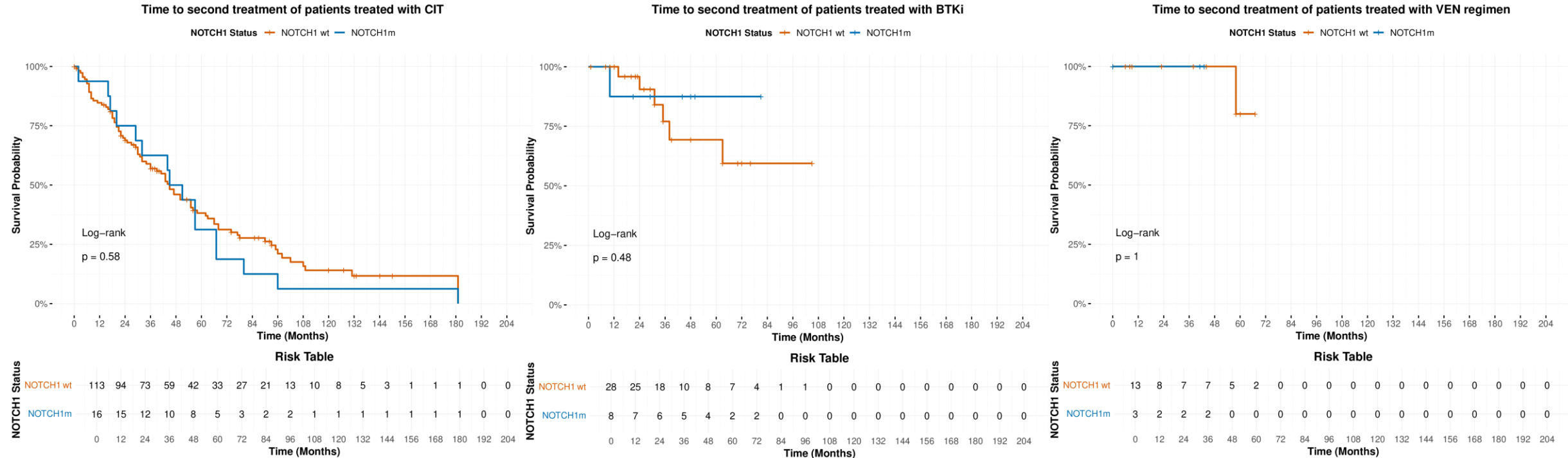

B

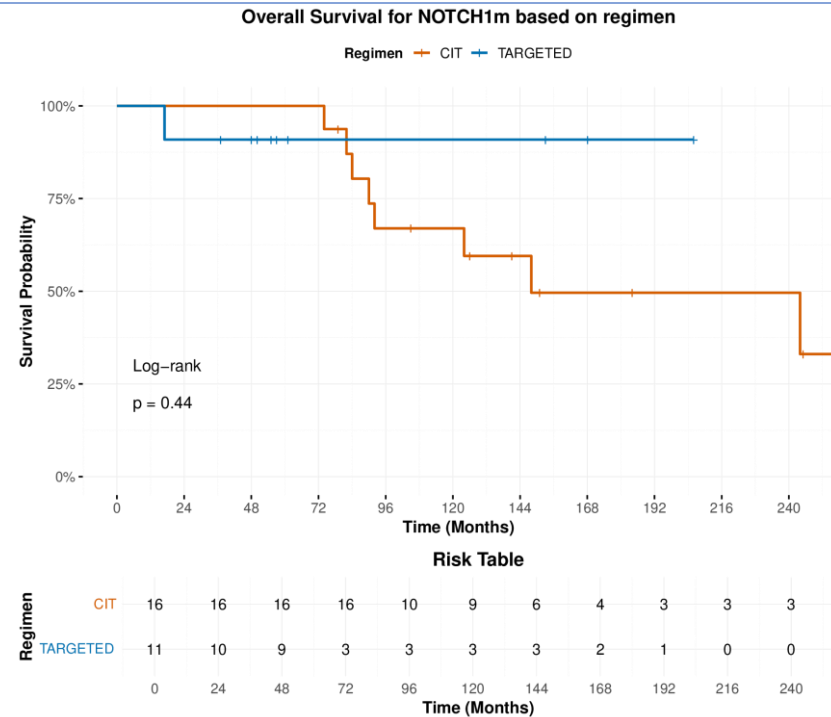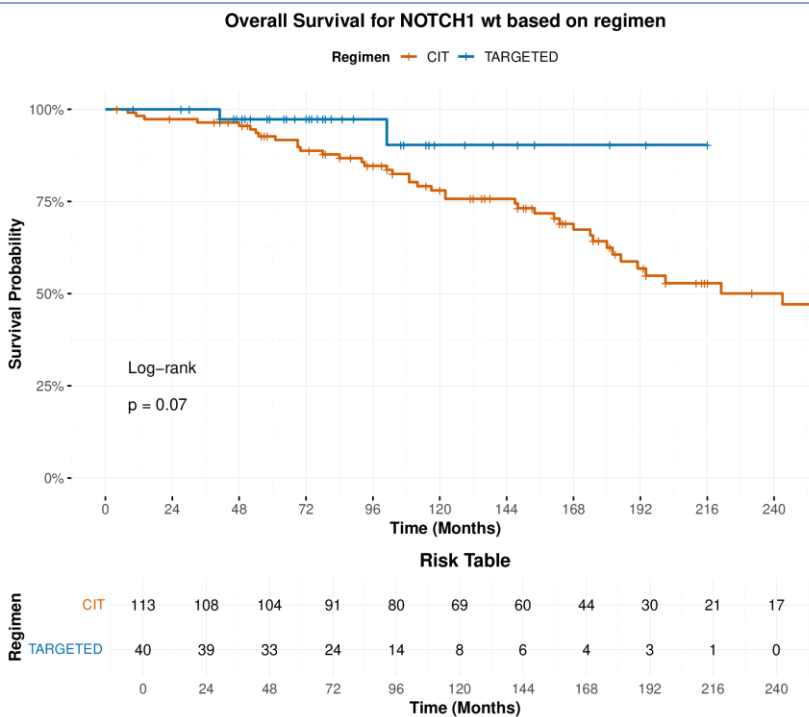

Supplement: Supplementary Figure 1 — (A) time to second treatment based on NOTCH1 status and first line regimen (B) Overall survival based on NOTCH1 mutation status and regimen. [file DataSheet1.pdf]
