# Supplementary material for: Prognostic and predictive impact of NOTCH1 mutations in patients with chronic lymphocytic leukemia: a tertiary single-center experience
Source: Front Oncol. 2026 Jan 13;15:1726439. doi: 10.3389/fonc.2025.1726439 (PMC12834786; doi:10.3389/fonc.2025.1726439)
Supplement: Supplementary file 2 [file DataSheet2.pdf]

Supplementary Table 1. Distribution of NOTCH1 mutations

NOTCH1 mutation	Frequency (n)	Percentage (%)
NOTCH1 p.P2514fs	32	84.2
NOTCH1 p.2515fs*4	3	7.9
NOTCH1 p.Q2460*	1	2.6
NOTCH1 p.L1601P	1	2.6
NOTCH1 p.L2482fs*1	1	2.6
**Totale**	**38**	**100.0**
