# Supplementary material for: Prognostic and predictive impact of NOTCH1 mutations in patients with chronic lymphocytic leukemia: a tertiary single-center experience
Source: Front Oncol. 2026 Jan 13;15:1726439. doi: 10.3389/fonc.2025.1726439 (PMC12834786; doi:10.3389/fonc.2025.1726439)
Supplement: Supplementary file 3 [file DataSheet3.pdf]

Supplementary Table 4. First-line treatment distribution by treatment era and NOTCH1 mutational status

	Pre-2015		≥2015	
Treatment type	NOTCH1 WT	NOTCH1 mut	NOTCH1 WT	NOTCH1 mut
Chemoimmunotherapy	63 (96.9%)	12 (100.0%)	49 (57.0%)	4 (26.7%)
Targeted therapy	2 (3.1%)	0 (0.0%)	37 (43.0%)	11 (73.3%)
**Total**	**65 (100%)**	**12 (100%)**	**86 (100%)**	**15 (100%)**

Distribution of first-line treatment types stratified by treatment era and NOTCH1 mutational status (n=178). Fisher's exact test for treatment distribution by era: $p<0.001$; by NOTCH1 status: $p=0.175$.
WT, wild-type; mut, mutated.
