# Supplementary material for: Prognostic and predictive impact of NOTCH1 mutations in patients with chronic lymphocytic leukemia: a tertiary single-center experience
Source: Front Oncol. 2026 Jan 13;15:1726439. doi: 10.3389/fonc.2025.1726439 (PMC12834786; doi:10.3389/fonc.2025.1726439)
Supplement: Supplementary file 4 [file DataSheet4.pdf]

Supplementary Table 3. Stratified analysis of NOTCH1 mutations by SHM status

Group	N	Events	HR (95% CI)	*P*-value
NOTCH1 wt / IGHV Mutated	101	13	1.00 (ref)	—
NOTCH1 wt / IGHV Unmutated	86	30	3.25 (1.65–6.40)	**<0.001**
NOTCH1m / IGHV Mutated	9	3	3.41 (0.95–12.23)	0.06
NOTCH1m / IGHV Unmutated	29	10	4.27 (1.83–9.96)	**<0.001**
Stratified analysis by SHM status				
NOTCH1 in IGHV Mutated	110	16	3.26 (0.89–11.94)	0.07
NOTCH1 in IGHV Unmutated	115	40	1.29 (0.63–2.65)	0.49

HR, hazard ratio; CI, confidence interval; SHM, somatic hypermutations;
IGHV, immunoglobulin heavy-chain variable region.
Analysis restricted to patients with complete NOTCH1 and SHM status data (n=225).
Global log-rank test p=0.0009.
Bold: $P < 0.05$.
