# Supplementary material for: Prognostic and predictive impact of NOTCH1 mutations in patients with chronic lymphocytic leukemia: a tertiary single-center experience
Source: Front Oncol. 2026 Jan 13;15:1726439. doi: 10.3389/fonc.2025.1726439 (PMC12834786; doi:10.3389/fonc.2025.1726439)
Supplement: Supplementary file 5 [file DataSheet5.pdf]

**Supplementary Table 2.** Cox regression analysis for overall survival: univariate, complete case, and multiple imputation

| | Univariate | | | Complete Case | | | MICE | | |
|---|---|---|---|---|---|---|---|---|---|
| Variable | HR | 95% CI | *P* | HR | 95% CI | *P* | HR | 95% CI | *P* |
| NOTCH1 mutations | 1.93 | 1.05-3.55 | **0.035** | 1.53 | 0.69-3.38 | 0.29 | 1.52 | 0.81-2.85 | 0.20 |
| Unmutated IGHV | 2.94 | 1.61-5.36 | **<0.001** | 2.48 | 1.22-5.03 | **0.012** | 2.56 | 1.42-4.62 | **0.003** |
| Therapeutic Era | 1.62 | 0.95-2.75 | 0.08 | 1.16 | 0.59-2.26 | 0.67 | 1.08 | 0.62-1.87 | 0.79 |
| TP53 mutations | 1.39 | 0.73-2.62 | 0.31 | 1.08 | 0.48-2.43 | 0.85 | 1.21 | 0.60-2.43 | 0.60 |
| Age at diagnosis | 1.11 | 1.08-1.14 | **<0.001** | 1.09 | 1.05-1.13 | **<0.001** | 1.10 | 1.07-1.14 | **<0.001** |
| Very complex Karyotype | 1.07 | 0.61-1.88 | 0.82 | 0.74 | 0.34-1.62 | 0.45 | 0.93 | 0.48-1.80 | 0.82 |
| *Interaction terms* | | | | | | | | | |
| NOTCH1 mutations × Therapeutic Era | — | — | — | 0.98 | 0.19-4.97 | 0.98 | 0.55 | 0.13-2.33 | 0.42 |

Abbreviations: HR, hazard ratio; CI, confidence interval; MICE, multiple imputation by chained equations.
Therapeutic Era: diagnosis/treatment after 2015
Complete case: $n$=180 patients (44 events). MICE: $n$=270 patients (67 events, 10 imputations).
C-index (complete case): 0.728. Bold: $P < 0.05$.
