# Supplementary material for: Prognostic and predictive impact of NOTCH1 mutations in patients with chronic lymphocytic leukemia: a tertiary single-center experience
Source: Front Oncol. 2026 Jan 13;15:1726439. doi: 10.3389/fonc.2025.1726439 (PMC12834786; doi:10.3389/fonc.2025.1726439)
Supplement: Supplementary file 6 [file DataSheet6.pdf]

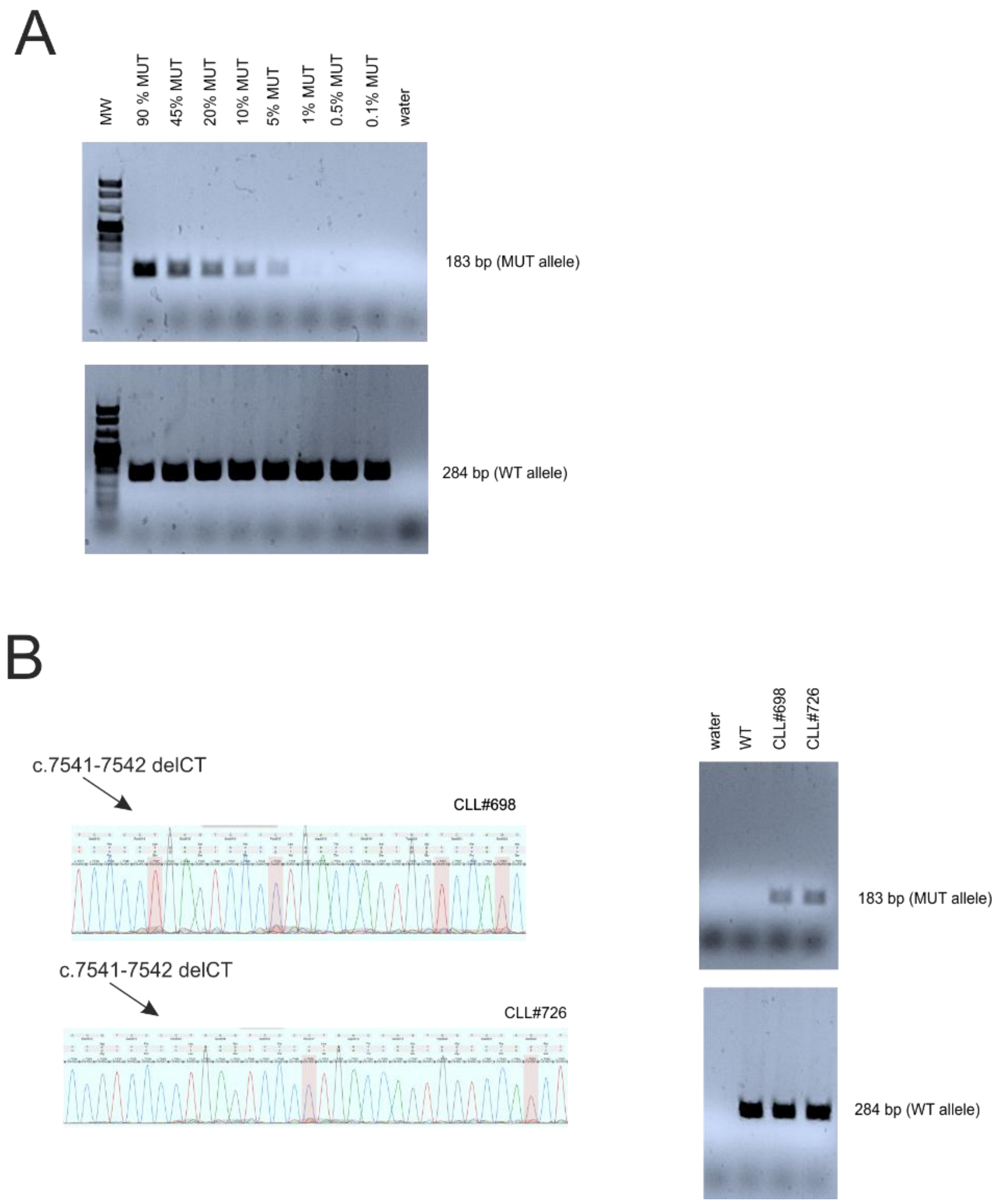

Supplementary Figure 2: (**A**) Representative results of the ARMS PCR assay showing the limit of detection for the c.7541_7542delCT mutation (MUT). DNA containing the mutant allele was progressively diluted with wild-type allele (WT). Wild-type allele shows a band of 284 bp, while the mutant band (MUT) is of 183 bp. Water control was also included. Molecular weight (MW) is the DNA molecular weight marker VIII. (**B**) Electropherogram showing the heterozygous CT deletion recurrently identified in CLL (left panels) in two representative CLL cases (#698, #726) with low mutational allele frequency and subsequently subjected to ARMS PCR for the NOTCH1 c.7541_7542delCT mutation (right panels). A wild-type (WT) sample and water control are also shown.
