# Supplementary material for: Prognostic and predictive impact of NOTCH1 mutations in patients with chronic lymphocytic leukemia: a tertiary single-center experience
Source: Front Oncol. 2026 Jan 13;15:1726439. doi: 10.3389/fonc.2025.1726439 (PMC12834786; doi:10.3389/fonc.2025.1726439)
Supplement: Supplementary file 7 [file DataSheet7.pdf]

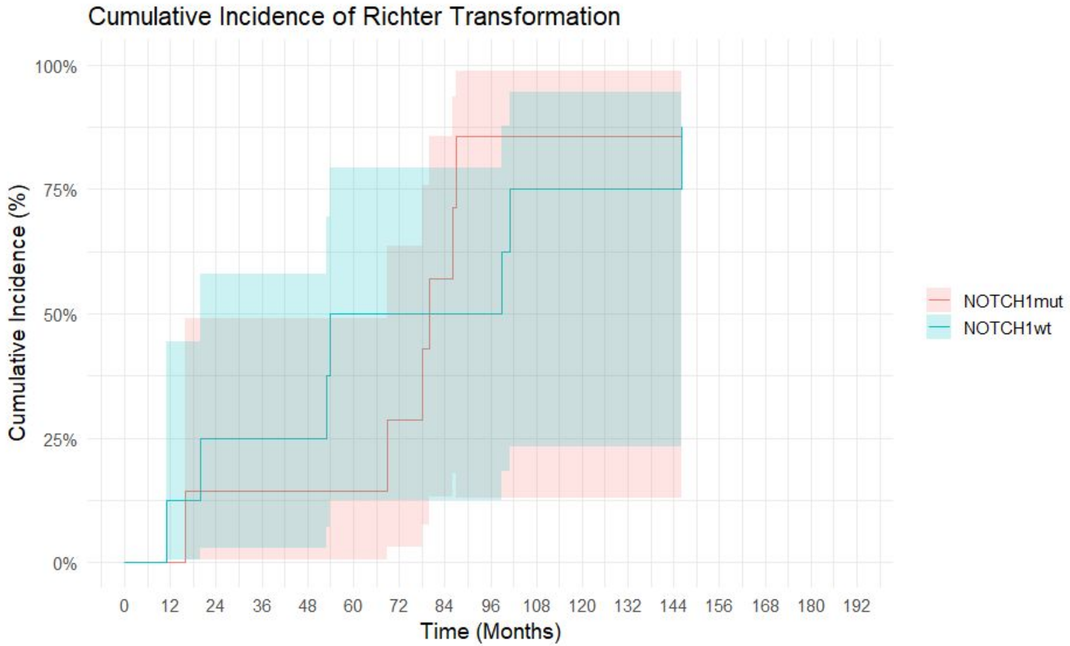

NOTCH1mut

At Risk	38	38	37	37	37	37	36	34	32	32	32	32	32	32	32	32	32
Events	0	0	1	1	1	1	2	4	6	6	6	6	6	6	6	6	6

NOTCH1wt

At Risk	233	232	231	231	231	229	229	229	229	227	227	227	227	226	226	226	226
Events	0	1	2	2	2	4	4	4	4	6	6	6	6	7	7	7	7

Supplementary Figure 3: cumulative incidence of Richter Transformation in NOTCH1 mutated and NOTCH1 wt patients
